# Supplementary material for: Corynebacterium glutamicum Regulation beyond Transcription: Organizing Principles and Reconstruction of an Extended Regulatory Network Incorporating Regulations Mediated by Small RNA and Protein–Protein Interactions
Source: Microorganisms. 2021 Jun 28;9(7):1395. doi: 10.3390/microorganisms9071395 (PMC8303971; doi:10.3390/microorganisms9071395)
Supplement: Supplementary file 1 [file microorganisms-09-01395-s001.zip › microorganisms-1063801-supplementary.pdf]

Supplementary material for

Corynebacterium glutamicum regulation beyond transcription: Organizing principles and reconstruction of an extended regulatory network incorporating regulations mediated by small RNA and protein-protein interactions

Juan M. Escorcia-Rodríguez [1], Andreas Tauch [2], and Julio A. Freyre-González [1,*]

[1] Regulatory Systems Biology Research Group, Laboratory of Systems and Synthetic Biology, Center for Genomic Sciences, Universidad Nacional Autónoma de México. Av. Universidad s/n, Col. Chamilpa, 62210. Cuernavaca, Morelos, México

[2] Centrum für Biotechnologie (CeBiTec). Universität Bielefeld, Universitätsstraße 27, 33615. Bielefeld, Germany

* Correspondence: jfreyre@ccg.unam.mx (J.A.F.G.)

Supplementary figures

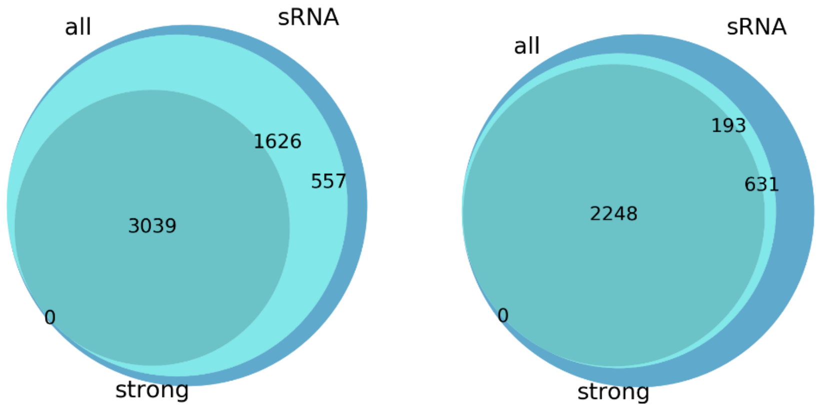

Supplementary Figure S1. The overlap between the tree network models of *C. glutamicum* for nodes (left) and interactions (right).

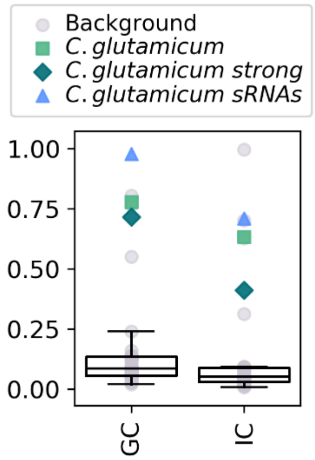

Supplementary Figure S2. Distribution of the genomic coverage (GC) and interactions coverage (IC) for the non-redundant set of regulatory networks from Abasy Atlas. The data points for the three *C. glutamicum* networks reported in this work are highlighted. Please note that the GC for the sRNA network is inflated by the sRNA nodes (545/3072) although there are still 86 protein-coding genes more than in the *all evidence* network.

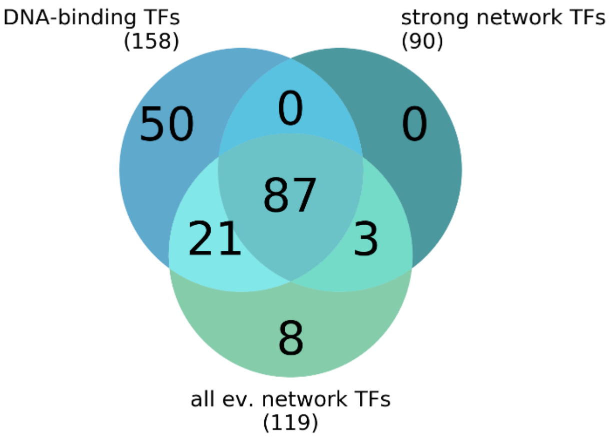

Supplementary Figure S3. Overlap of transcription factors between the two transcriptional regulatory networks and the complete set of DNA-binding transcription factors in *C. glutamicum*.

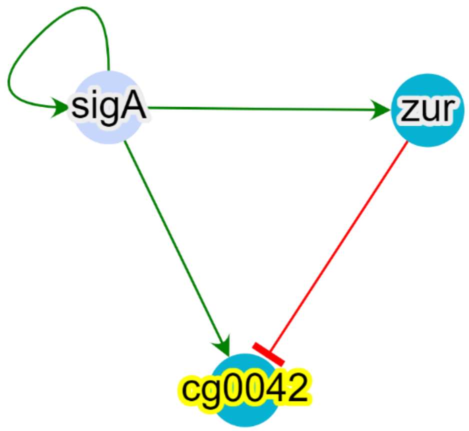

Supplementary Figure S4. Interaction *zur-cg0042* part of the *C. glutamicum strong* network also recovered from *S. coelicolor*.

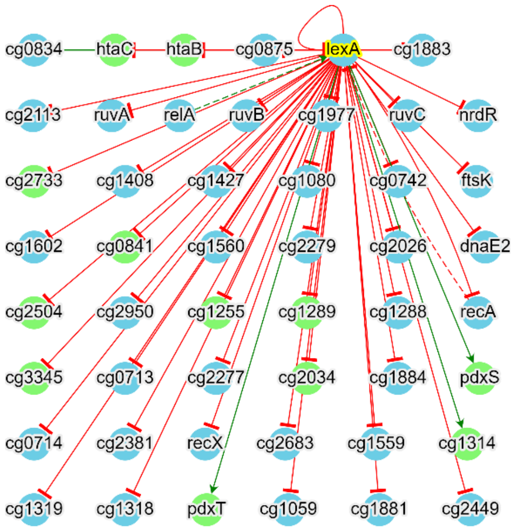

Supplementary Figure S5. LexA auto-regulation part of the *C. glutamicum* network strongly supported and recovered from *B. subtilis*.

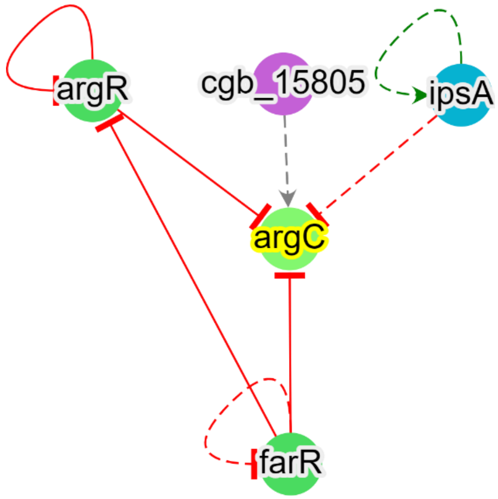

Supplementary Figure S6. Interaction *argR-argC* part of the *C. glutamicum* strongly supported and recovered from *E. coli*.

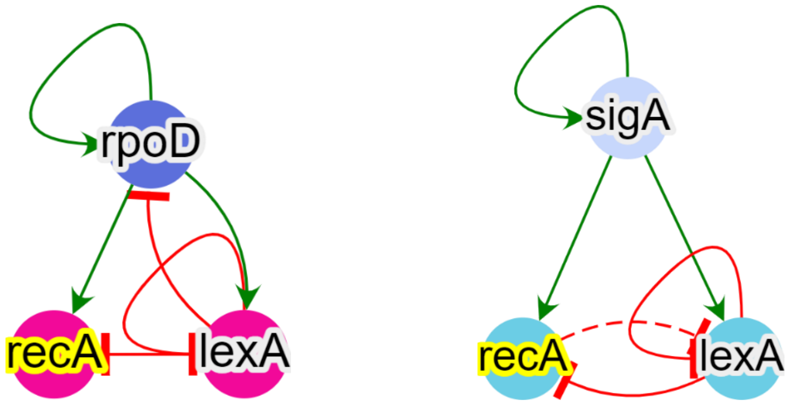

Supplementary Figure S7. The interaction *lexA-recA* was recovered from *E. coli* (left) and is already part of the *C. glutamicum* network (right) strongly supported.

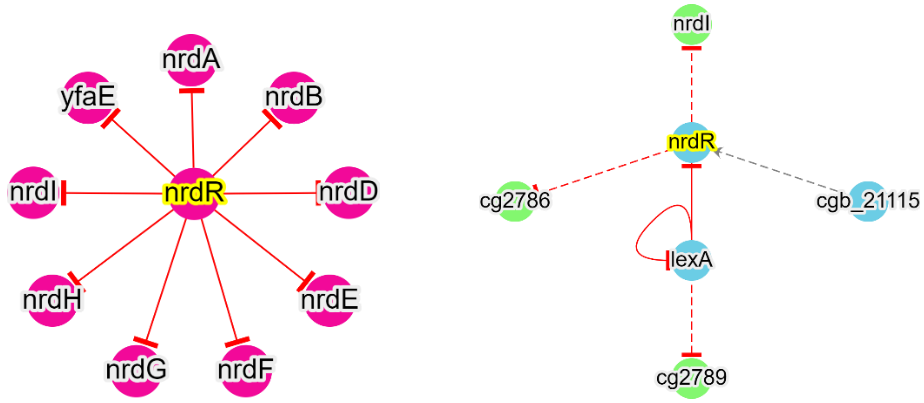

Supplementary Figure S8. The interaction *nrdR-nrdI* was recovered from *E. coli* (left) and is already part of the *C. glutamicum* network (right) with non-strong evidence supporting it.

Supplementary tables

Supplementary Table S1. Interactions from other *C. glutamicum* strains or not mapping to a cg number. Please consider this is not an exhaustive list of regulations for other strains.

TF	TG	Effect
cg3247	*NCgl2845*	+
cg3247	*NCgl1729*	+
cg0702	*cg1652*	-
cg0702	*cg1583*	-
cg0702	*cg3378*	-
cg0702	*cg0400*	+
cg0702	*NCgl0166*	+
cg0702	*cg1582*	-
cg0702	*cg3387*	+
cg0702	*NCgl0484*	+
cg0702	*cg0771*	+
cg0702	*cg3327*	-
cg3224	*NCgl1861*	-
cg0702	*cg0344*	+
cg3247	*NCgl2113*	+
cg0702	*NCgl2942*	+
cg0702	*NCgl2893*	+
cg0702	*cg1215*	-
cg0702	*cg3375*	+
cg0702	*NCgl0746*	-
cg0146	*NCgl18*	+
cg0702	*cg1528*	-
cg0702	*NCgl0608*	+
cg0702	*cg2252*	+
cg3224	*NCgl2817*	-
cg0702	*cg0345*	+
cg0702	*cg1581*	-
cg2831	*NCgl1806*	+
cg3224	*NCgl0361*	+
cg0702	*cg1584*	-
cg3247	*NCgl2738*	-
cg3247	*NCgl2237*	-
cg0702	*NCgl0580*	+
cg3247	*NCgl2861*	+
cg0702	*NCgl2970*	+
cg0702	*cg0661*	-
cg3247	*NCgl1758*	+

cg0702	*cg0018*	+
cg3247	*NCgl0404*	+
cg0702	*cg1214*	-
cg3224	*NCgl0360*	+
cg3247	*NCgl2858a*	+
cg3247	*NCgl1780*	+
cg0702	*cg1120*	+
cg0702	*cg3145*	+
cg0702	*cg0197*	+
cg0702	*cg0318*	+
cg2831	*NCgl1812*	-
cg2831	*NCgl1783*	+
cg3247	*NCgl1750*	+
cg0702	*NCgl0638*	+

Supplementary Table S2. Interactions recovered from *S. coelicolor, B. subtilis,* and *E. coli*. The *Average rank* is the averaged ranking position between the three inferences with the three motif finding tools (smaller is better). The *Status* can take three values: 1) *network* for interactions that have been already experimentally validated and are part of one of the *C. glutamicum* networks; 2) *potential new TG* for the interactions mediated by a TF that is already a TF in one of the networks, but its regulation of the TG expression has not been experimentally validated; and 3) *new TF* for interactions mediated by TFs with uncharacterized regulons, hence those that are not included in the current networks.

TF	TG	Average rank	Status
Recovered from *S. coelicolor*			
cg0484	*cg2261*	10.0	new TF
cg1585	*cg0850*	42.333	potential new TG
cg2502	*cg0042*	50.0	network
cg1585	*cg2305*	51.0	potential new TG
cg3202	*cg2117*	54.333	potential new TG
cg0484	*cg2846*	57.333	new TF
cg2502	*cg0991*	59.333	potential new TG
cg0484	*cg1809*	61.333	new TF
cg3202	*cg2929*	62.0	potential new TG
cg1585	*cg0113*	63.0	potential new TG
cg0484	*cg2260*	65.333	new TF
cg1486	*cg1487*	67.0	network
cg0484	*cg2485*	67.333	new TF
cg1585	*cg1580*	68.0	network
cg2109	*cg2109*	69.0	network

cg1585	*cg0303*	71.0	potential new TG
cg1486	*cg2383*	71.0	potential new TG
cg1585	*cg1586*	72.0	potential new TG
cg1585	*cg2261*	73.333	potential new TG
cg1098	*cg1098*	74.333	new TF
cg0484	*cg2513*	76.667	new TF
cg0313	*cg0313*	77.0	network
cg1486	*cg1486*	83.667	potential new TG
cg3202	*cg3202*	85.667	network
Recovered from *B. subtilis*			
cg2114	*cg2114*	3.333	network
cg2114	*cg2141*	11.667	network
cg2114	*cg1996*	15.333	potential new TG
cg2624	*cg2803*	25.0	potential new TG
cg1585	*cg1580*	27.333	network
cg2624	*cg1142*	29.667	potential new TG
cg1585	*cg1582*	39.333	network
cg2516	*cg3100*	39.667	potential new TG
cg3097	*cg0113*	40.0	potential new TG
cg1817	*cg1814*	40.667	potential new TG
cg1817	*cg1815*	42.333	network
cg2516	*cg2514*	44.667	potential new TG
cg2114	*cg1401*	47.333	potential new TG
cg1817	*cg1817*	48.0	network
cg2516	*cg3099*	49.667	potential new TG
cg2114	*cg1560*	49.667	network
cg2114	*cg0976*	50.667	potential new TG
Recovered from *E. coli*			
cg1585	*cg1580*	19.0	network
cg2114	*cg2114*	62.667	network
cg2114	*cg2141*	64.0	network
cg0350	*cg2166*	115.333	potential new TG
cg0350	*cg3395*	164.333	potential new TG
cg2899	*cg2637*	169.0	new TF
cg3224	*cg2559*	190.667	potential new TG
cg0350	*cg1568*	195.0	network
cg1585	*cg3004*	201.0	potential new TG
cg0350	*cg2429*	209.667	network
cg0350	*cg1257*	211.333	potential new TG
cg0350	*cg0229*	212.0	network
cg0001	*cg0001*	215.667	new TF
cg1327	*cg1327*	217.0	new TF

cg2502	*cg0591*	224.0	potential new TG
cg0350	*cg2175*	229.667	potential new TG
cg0350	*cg2126*	232.0	potential new TG
cg1585	*cg2167*	235.0	potential new TG
cg0350	*cg3068*	243.667	potential new TG
cg0350	*cg2870*	243.667	potential new TG
cg0350	*cg1145*	244.0	network
cg1327	*cg3141*	250.667	new TF
cg2502	*cg2782*	253.667	potential new TG
cg2936	*cg2933*	254.333	network
cg2502	*cg3237*	255.0	potential new TG
cg0350	*cg3308*	256.0	potential new TG
cg0350	*cg2102*	263.0	potential new TG
cg0350	*cg1492*	266.667	potential new TG
cg0001	*cg1525*	273.667	new TF
cg2112	*cg2787*	281.667	network
cg1425	*cg0001*	283.333	potential new TG
cg2502	*cg2183*	285.0	potential new TG
cg0350	*cg2841*	286.0	potential new TG
cg1585	*cg2166*	287.333	potential new TG
cg0350	*cg1790*	290.333	network
cg0350	*cg3340*	290.667	potential new TG
cg2502	*cg2502*	291.0	potential new TG
cg0350	*cg3423*	291.0	potential new TG
cg0350	*cg1586*	293.0	potential new TG
cg1327	*cg1656*	293.667	new TF
cg1585	*cg2178*	298.0	potential new TG
cg1585	*cg0229*	299.0	network
cg0350	*cg0350*	299.0	network
cg0350	*cg0067*	300.667	potential new TG
cg2114	*cg2489*	303.0	potential new TG
cg0350	*cg0953*	304.333	potential new TG
cg1425	*cg0004*	309.0	potential new TG
cg1585	*cg1588*	310.0	potential new TG
cg2112	*cg2781*	313.0	potential new TG
cg1585	*cg1586*	314.333	potential new TG
cg1585	*cg1814*	317.333	network
cg1327	*cg1355*	320.0	new TF
cg2112	*cg2789*	327.667	network
cg0001	*cg0004*	328.0	new TF
cg1327	*cg2856*	329.0	new TF
cg1425	*cg0306*	331.0	potential new TG

cg2899	*cg2899*	331.667	new TF
cg0350	*cg0699*	333.667	potential new TG
cg1425	*cg0005*	334.667	potential new TG
cg0350	*cg0673*	340.667	potential new TG
cg0350	*cg3336*	345.333	potential new TG
cg0350	*cg1163*	351.667	potential new TG
cg1327	*cg1383*	362.333	new TF
cg0350	*cg2932*	362.667	potential new TG
cg0001	*cg1550*	370.333	new TF
cg0001	*cg0005*	374.333	new TF
cg2114	*cg1602*	375.0	network
cg1327	*cg2891*	376.667	new TF
cg0350	*cg2178*	379.0	potential new TG
cg0350	*cg3096*	379.667	network
